# Nobiletin enhances the efficacy of chemotherapeutic agents in ABCB1 overexpression cancer cells

**DOI:** 10.1038/srep18789

**Published:** 2015-12-22

**Authors:** Wenzhe Ma, Senling Feng, Xiaojun Yao, Zhongwen Yuan, Liang Liu, Ying Xie

**Affiliations:** 1 State Key Laboratory of Quality Research in Chinese Medicine, Macau Institute For Applied Research in Medicine and Health, Macau University of Science and Technology, Avenida Wai Long, Taipa, Macau (SAR), China

## Abstract

Multidrug resistance (MDR) is the major obstacle to the successful chemotherapy treatment of many cancers. Here we found that nobiletin, a citrus methoxyflavone, significantly sensitized ABCB1 overexpressing cells A2780/T and A549/T to chemotherapeutic agents such as paclitaxel (a 433-fold reversal of MDR to PTX at 9 μM), doxorubicin (DOX), docetaxel and dounorubicin. Nobiletin profoundly inhibited ABCB1 transporter activity since it significantly increased the intracellular accumulation of DOX and Flutax-2 in A2780/T cells and decreased the efflux of ABCB1 substrates in Caco2 cells without altering the mRNA and protein expression of ABCB1. Moreover, nobiletin stimulated ATPase activity and inhibited verapamil-stimulated ATPase activity in a concentration-dependent manner, indicating a direct interaction with the transporter. Consistent with these findings, molecular docking analysis also identified favorable binding of nobiletin with the transmemberane region site 1 of homology modeled human ABCB1 transporter. Moreover, the Nrf2 protein expression and phosphorylation levels of AKT/ERK were suppressed by co-treated with nobiletin and PTX at the reversal concentrations, suggesting that inhibition of the AKT/ERK/Nrf2 pathway was associated with the sensitizing effect of nobiletin. These findings encourage further animal and clinical MDR studies with the combination therapy of nobiletin and chemotherapeutic drugs.

Multi-drug resistance (MDR) is the major reason for the clinical failure of many forms of chemotherapy^1^. In the past few decades, a number of different mechanisms were found to mediate the development of MDR, and the most important ones were those which associated with overexpression of various members of the ATP binding cassette (ABC) transport proteins^2^,^3^. The human ABCB1 (MDR1)-encoded multidrug transporter P-glycoprotein (P-gp) is the most extensively studied ABC transporter^4^,^5^, which is significantly elevated in drug-resistant tumors, pumping out various anticancer drugs, such as taxanes, anthracyclines, *vinca* alkaloids, and epipodophyllotoxins^1^. Since 1981, P-gp inhibitors have been intensively studied as potential MDR reversers^6^. Though several P-gp inhibitors were found among the available drugs, their toxicity and drug interaction profiles drove researchers to search for new, more effective compounds with low toxicity and fewer side effects^7^.

Moreover, recently studies showed that activation of PI3K/AKT, ERK and Nrf2 pathways were associated with resistance to chemotherapeutic drugs^8^,^9^,^10^. Antitumor drugs are known to inhibit these signaling pathways and consequently increase tumor cell sensitivity to chemotherapy drugs^11^,^12^. Therefore, identification of inhibitors that potently inhibit the activation of AKT/ERK and Nrf2-denpendent response is desirable for reversing MDR. Currently, researches are stepping toward natural products as potential MDR reversers since they are safe and non-toxic^13^,^14^.

Nobiletin (Fig. 1A) is a non-toxic dietary polymethoxylated flavone and present in some citrus fruits such as *Citrus depressa* (shiikuwasa) and *Citrus sinensis* (oranges)^15^,^16^. It was reported to exhibit multiple biological effects such as anti-inflammatory, anti-tumor, and neuroprotective properties^17^,^18^,^19^. As a potent chemo-preventive agent, nobiletin inhibited the growth of several prostate cancer cell lines with IC_50_ values around 100 μM by causing cell cycle arrest in G_0_/G_1_ phase^20^,^21^,^22^. Moreover, it has been reported that nobiletin could increase accumulation of daunorubicin in KB-C2 cells at 50 μM^23^ and the uptake of [^3^H] vinblastine in Caco-2 cells^24^ as well as in ABCB1 transfected LLC-GA5-COL300 cells^24^,^25^ at 20 μM, indicating the potential P-gp inhibition effect of nobiletin. However, whether and to what extent nobiletin inhibits P-gp in MDR cancer cell lines, and whether this activity contributes to MDR reversal are still elusive.

In this study, we performed a series of experiments to investigate the reversal effect of nobiletin on ABCB1 overexpressing cancer cell lines to chemotherapeutic agents including paclitaxel (PTX), doxorubicin (DOX), docetaxel and dounorubicin. Nobiletin at achievable nontoxic plasma concentrations (0.5 to 9 μM)^26^ significantly sensitizes the ABCB1 overexpressing MDR cancer cell lines by modulating the ABCB1 function and inhibiting the AKT/ERK/Nrf2 pathways, therefore, has the potential to be used in combination therapies to treat MDR.

## Results

### Demonstration of multidrug resistance in cell line model

**Figure 1:**
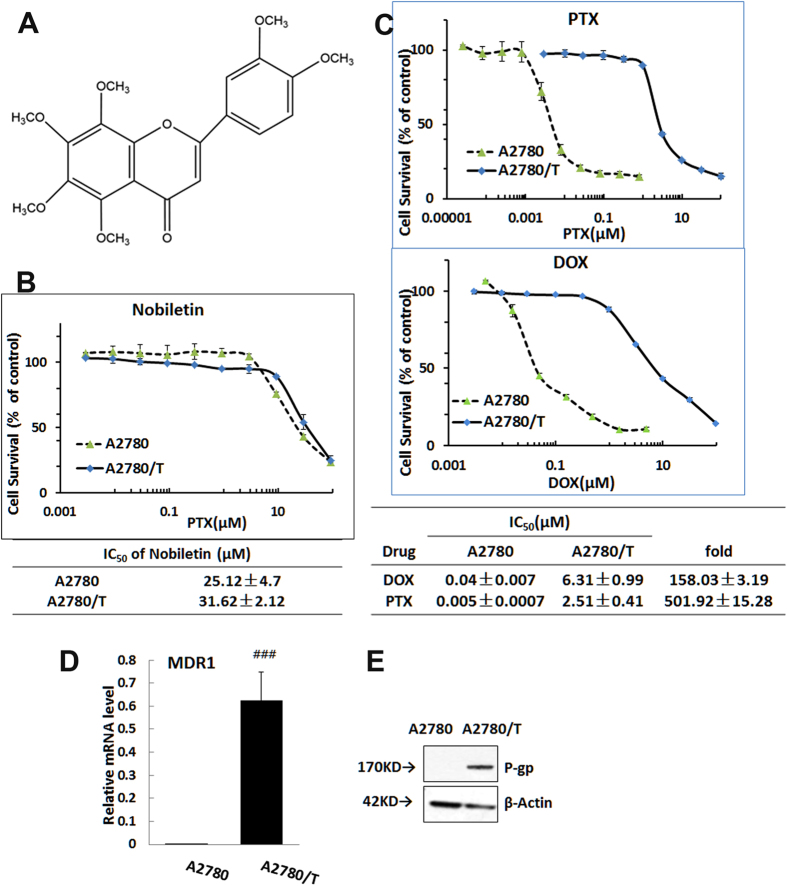
Demonstration of multidrug resistance in PTX- resistant ovarian cancer cells (A2780/T). (**A**) Chemical Structures of nobiletin. (**B**) Cytotoxicity of nobiletin alone in pairs of A2780/T or A2780 cells. (**C**) The cells were treated with various concentrations of paclitaxel (PTX) and doxorubicin (DOX) for 48 hours. Cell growth was determined using the SRB assay. The expression of ABCB1 transporter in A2780 and A2780/T cells was analyzed at level of both *MDR1* mRNA by RT-qPCR (**D**) and P-gp protein level by Western blotting (**E**). (^###^ Significantly different from A2780 cells with P < 0.001). Protein expression levels after normalized relatively to that of β-actin.

We determined the IC_50_ values of several anti-cancer drugs in a stably paclitaxel-resistant cell line (A2780/T) and its parental line (A2780). The mean IC_50_ values for PTX and DOX were 501-fold and 158- fold greater in A2780/T cells than that of A2780 (Fig. 1C), which confirmed that this cell line exerted much higher tolerance than the parental sensitive cell line. In Fig. 1D,E, RT-qPCR and Western blot analysis confirmed that the *MDR1* gene and P-gp protein in the A2780/T were all significantly higher than that of A2780 cells (*p* < 0.01).

### Nobiletin sensitizes ABCB1-overexpressing cells to chemotherapeutic agents

Firstly, the intrinsic cytotoxicity of nobiletin was measured in A2780 and A2780/T as well as A549 and A549/T by the SRB assay. Nobiletin has similar IC_50_ for both A2780 and A2780/T (without adding 0.94 μM PTX to culture medium) (Fig. 1B) as well as similar values for both A549 and A549/T cells (See Supplementary Fig. S1A). Notably, the results showed that nobiletin at 9 μM, had no obvious cytotoxic effect to all cell lines, and more than 90% cells were viable. Based on these data, nobiletin was tested in the reversal assays at the maximum concentration of 9 μM.

**Figure 2:**
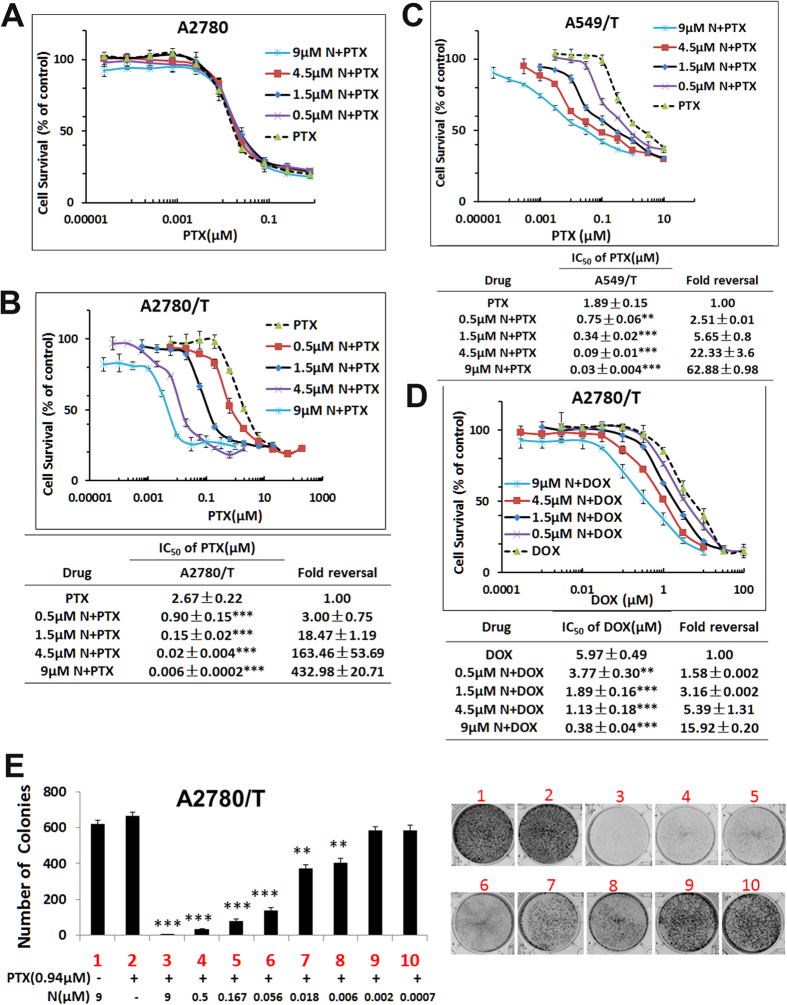
Effect of nobiletin on reversing ABCB1- mediated resistance. Cells were treated with the indicated drugs for 48 hours and subjected to SRB assay. Nobiletin reduces the IC_50_ of paclitaxel in resistant cancer cells (A2780/T) (**B**) but not in drug sensitive (A2780) (**A**). (**C**) Nobiletin reduces the IC_50_ of paclitaxel in resistant NSCLC cancer cells (A549/T). (**D**) Nobiletin reduces the IC_50_ of doxorubicin in resistant cancer cells (A2780/T). (**E**) Nobiletin inhibited the colony formation of paclitaxel in resistant cancer cells A2780/T in a dose-dependent manner. ^##^ or ^**^P < 0.01, ### or ***P < 0.001, significantly different from those obtained in the absence of nobiletin.

Next, we tested whether nobiletin could reverse the MDR of A2780/T cells. Treatment with nobiletin significantly decreased the IC_50_ of PTX and DOX in A2780/T cell in a concentration-dependent manner, as shown by the shift in the cytotoxicity curves to the left in Fig. 2B,D. Specifically, treatment with 0.5, 1.5, 4.5, and 9 μM nobiletin reduced the IC_50_ of PTX by 3.0-, 18.5-, 163.5-, and 432.9- fold, respectively, in A2780/T cells. The IC_50_ of DOX was decreased 1.58-, 3.16-, 5.39- and 15.92-fold after combination treatment with 0.5, 1.5, 4.5, and 9 μM nobiletin, respectively. However, nobiletin, at tested concentrations, had no effect on the IC_50_ of PTX and DOX in the parental sensitive A2780 cells (Fig. 2A, and Supplementary Fig. S1C). Moreover, at the concentration of 4.5 μM, nobiletin also reduced the IC_50_ values of docetaxel and daunorubicin with reversal fold of 15.8 and 13.6, respectively, whereas it also slightly decreased the IC_50_ values of 5-fluorouracil (non-substrate of ABCB1) with a reversal fold of 6.3 as shown in table 1.

In another ABCB1-overexpressing non-small cell human lung cancer cell line A549/T (PTX-resistance) and its parental cells A549, we observed similar reversal effects of nobiletin to PTX and other chemotherapeutic agents (Supplementary Table S1). In Fig. 2C, the addition of nobiletin at 0.5, 1.5, 4.5, and 9 μM significantly decreased the IC_50_ of PTX with reversal fold of 2.51, 5.65, 22.33 and 62.88, respectively. At same concentrations, nobiletin had no effect on the IC_50_ of PTX in the parental sensitive A549 cells (Supplementary Fig. S1B). All these results suggested that nobiletin significantly sensitized ABCB1-overexpressing cells to chemotherapeutic drugs that are substrates of ABCB1.

In order to confirm whether the drug sensitization effect is related to the specific transport protein ABCB1, we also tested the effect of quinidine (QND, the inhibitor of P-gp), MK571 (the inhibitor of MRPs) and KO143 (the inhibitor of BCRP) on sensitizing A2780/T cells to low-dose PTX-induced death. The fold-reversal of QND at concentration of 0.24, 0.72 and 2.16 μM to PTX was 3.25, 43.15, and 252.83, respectively, in A2780/T cells (See Supplementary Fig. S2A). However, QND showed an intimal cytotoxicity at concentration of 6.48 μM with only 40% viable cells. Moreover, MK571 and KO143 had no effects on the IC_50_ of PTX which demonstrated that the reversal effect was specific to ABCB1 transporter (Supplementary Fig. S2B).

The long term reversal effects of nobiletin on ABCB1 mediated MDR to PTX were further evaluated using colony formation assays. Complete inhibition of colony formation was achieved with the combination of 0.94 μM PTX and different concentrations of nobiletin, whereas no inhibition was observed at either 9 μM nobiletin or 0.94 μM PTX alone (Fig. 2E). And with different concentration below 0.5 μM, nobiletin could inhibit the A2780/T cells colony formation in a dose-dependent manner. Taken together, these results indicated that combination of nobiletin with PTX elicited significantly higher cytotoxic response in ABCB1 overexpression MDR cancer cells.

### Nobiletin potentiates PTX induced apoptosis in resistant A2780/T cells

**Figure 3:**
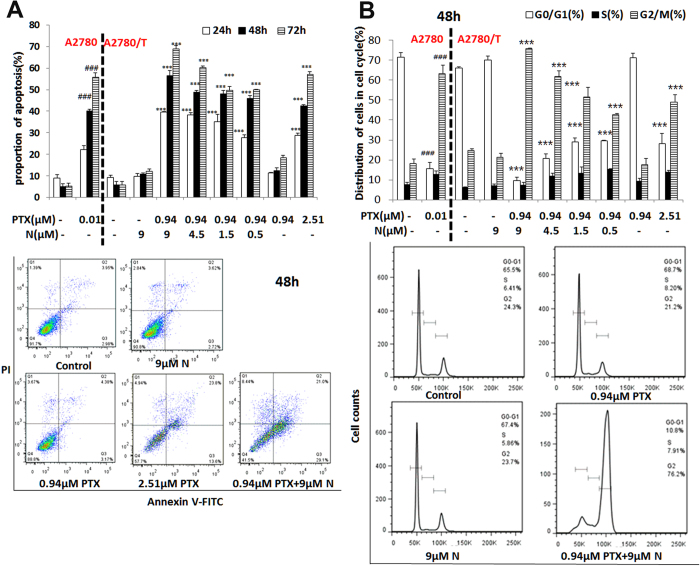
Effect of the combination treatment with nobiletin and 0.94 PTX on the apoptosis and cell cycle of MDR cancer cells. (**A**) The cells were treated with different concentrations of nobiletin and/or PTX (0.94 μM) for 48 h, stained with annexin V–FITC, and analyzed by flow cytometry. (**B**) The cell cycle distribution profiles of the cells treated with nobiletin and/or PTX (0.94 μM) were determined by flow cytometry. The data are representative of three different experiments and are shown as mean ± SD (n = 3). ^##^ or **P < 0.01, ^###^ or ***P < 0.001, significantly different from the control group.

We next investigated whether nobiletin increased the PTX-induced apoptosis in A2780 and A2780/T cells using flower cytometry. Consistent with its ability to inhibit cell growth, treatment with 0.5, 1.5, 4.5, and 9 μM nobiletin significantly increased apoptosis induced by 0.94 μM PTX in a concentration-dependent manner, as shown in Fig. 3A. We found that treatment with only 0.5 μM nobiletin could boost the apoptosis induced by PTX (0.94 μM) to a similar degree as that of 2.51 μM PTX (IC_50_ of A2780/T). While single treatment of 9 μM nobiletin or 0.94 μM PTX did not show apoptosis induction.

Since it has been recently reported that nobiletin also caused G2/M cell cycle arrest^27^, we then evaluated whether this is related to our observed synergistic effect between nobiletin and PTX. Asynchronously growing A2780/T cells and its sensitive parental cell line A2780, both treated with PTX in the absence or presence of nobiletin, were examined for their cell cycle progressions by flow cytometry. In untreated control, the percentage of A2780 cells in G_0_/G_1_-, S- and G_2_/M- phases were 71.6%, 7.76% and 18.27%, respectively, the percentage of A2780/T cells in G_0_/G_1_-, S- and G_2_/M- phases were 66.13%, 6.25% and 24.77%, respectively. Single exposure for 24, 48, and 72 hours to PTX (0.01 μM) resulted in G2-M arrest in A2780 cells (Fig. 3B).

In the absence of nobiletin treatment, there were 72% of cells at G1 phase and 17% of cells at G2 phase after incubated with 0.94 μM PTX, whereas these distributions were significantly shifted to 9.6% of G1 and 75.77% of G2 phase cells after treatment of nobiletin at 9 μM in combination with 0.94 μM PTX (Fig. 3B). This pattern was evident after 24 h and persisted over the 72 h of treatment. As shown in Fig. 3B, a notable G2/M arrest was observed even with the lowest concentration of nobiletin tested (0.5 μM). Thus, while A2780/T cells were remarkably resistant to 0.94 μM PTX, the combination of nobiletin with PTX was found to greatly increase the proportion of G2/M arrested cells to >75%. However nobiletin (9 μM) alone had no effect on cell cycle distribution of A2780/T.

**Figure 4:**
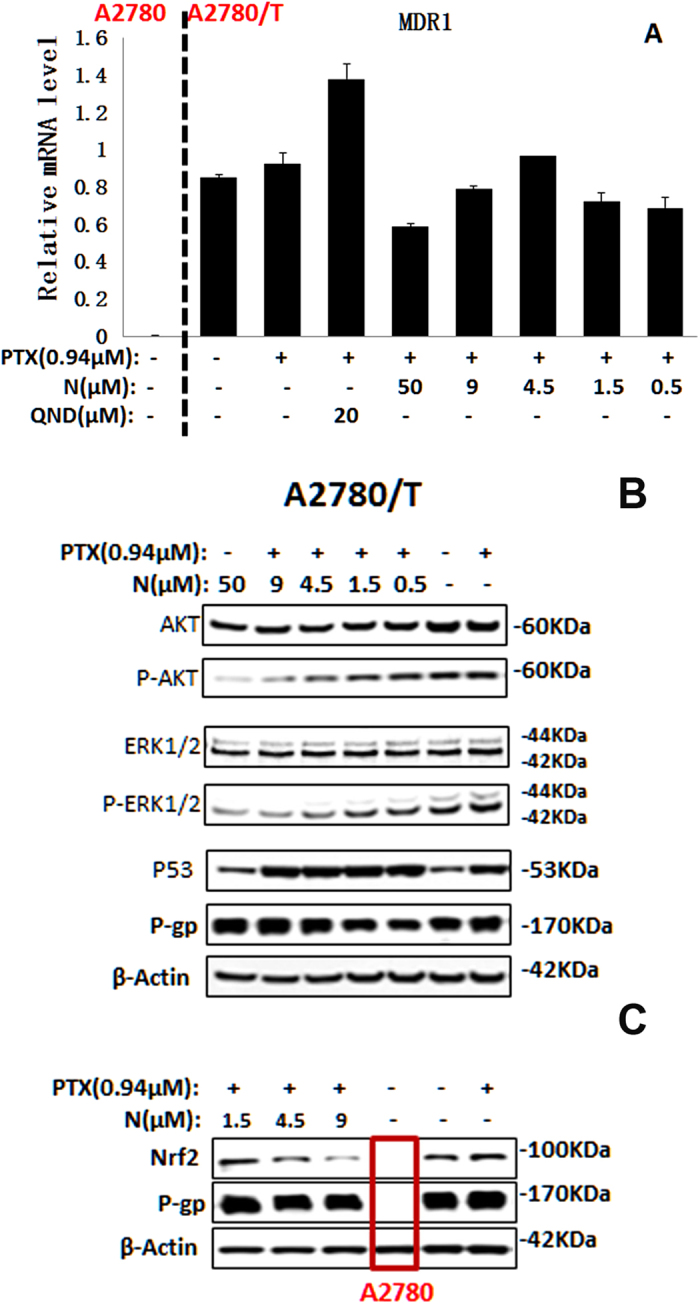
Effects of the combination treatment of paclitaxel and nobiletin on ABCB1 expression and AKT/ERK/Nrf2 pathway. (**A**) The *MDR1* mRNA level was determined by RT-PCR in A2780/T cells or A2780 cells. Combination treatment of paclitaxel and nobiletin did not influence P-gp expression levels, but unregulated the p53 expression (**B**) and reduced the expression level of Nrf2 (**C**) as well as the phosphorylation of AKT/ERK (**B**) by Western blot assay.

To further confirm these results, we examined the well-established biochemical markers of cell cycle arrest and apoptosis: p53. Consistent with cell growth inhibition and apoptosis, treatment of PTX in combination with nobiletin resulted in accumulation of p53 in treated cells (Fig. 4B).

### Nobiletin exerts synergistic effect combining with PTX in MDR cells

The combination cytotoxic effect of nobiletin with PTX in A2780/T cells was further evaluated using the Median Effect method described by T-C Chou and P. Talalay^28^. The combination index (CI) values calculated at 50% (ED_50_) and 90% (ED_90_) of cell killing were 0.013 and 5.14 × 10^−5^ as shown in supplementary table S2, demonstrating a very strong synergistic cytotoxic effect (CI < 0.1) of the nobiletin-PTX combination in the ABCB1-overexpressing A2780/T cells. With CalcuSyn simulation, an ED_50_ is produced by 35.96 μM of nobiletin or 4.20 μM of PTX in A2780/T cells, but the ED_50_ of PTX is 0.022 μM in combined with 0.286 μM nobiletin which is a 200-fold decrease for the ED_50_ value (Supplementary Table S2). The quantitative diagnostic graphics for this synergistic effect between nobiletin and PTX are shown in supplementary Fig. S3.

### Nobiletin increases the intracellular accumulation of DOX and Flutax-2

The above results proved that nobiletin have a significant effect on reversing ABCB1-mediated MDR. At present, the mechanism of this phenomenon is unknown. Therefore, we conducted following assays to examine the effect of nobiletin on the accumulation of DOX, and Flutax-2 (a fluorescent taxol derivative) in A2780 cells and their corresponding ABCB1-overexpressing A2780/T cells using fluorescence microscope and flow cytometry analysis.

**Figure 5:**
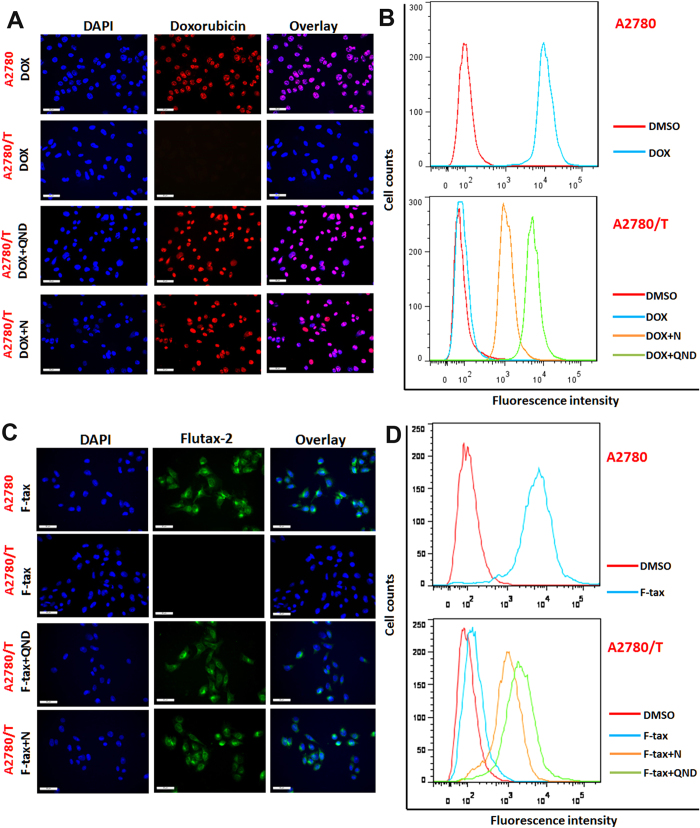
Effect of nobiletin on intracellular accumulation of doxorubicin (DOX) and flutax-2(F-tax) in MDR ovarian cancer cells. A2780 cells or A2780/T Cells treated with 5 μM DOX (**A**,**B**) or 1 μM F-tax (**C**,**D**) for 8 hours in the absence or presence of 4.5 μM nobiletin, and 20 μM quinidine (positive control) as indicated. Intracellular DOX and F-tax accumulation were observed with a florescence microscope (**A**,**C**) and evaluated by measuring florescence with flow cytometry (**B**,**D**) as described in Method. The experiments were repeated for at least 3 times, presented are representative images.

The intracellular accumulation of DOX and Flutax-2 were significantly higher in A2780 than that in A2780/T (Fig. 5). When the drug-resistant cells were treated with 4.5 μM nobiletin or 20 μM QND (positive control), the intracellular accumulation of DOX (Fig. 5A), and Flutax-2 (Fig. 5C) were higher than that in untreated A2780/T. In contrast, nobiletin had no effect on DOX and Flutax-2 levels in the parental A2780 cells. Enhanced intracellular accumulation of DOX, or Flutax-2 by nobiletin was further confirmed by flow cytometry analysis as shown in Fig. 5B,D. Taken together, these results showed that nobiletin significantly increased the intracellular accumulation of chemotherapeutic drugs in ABCB1-overexpressing cells, thus increasing the cytotoxicity in these MDR cells.

### Nobiletin inhibits the efflux activity of ABCB1 transporter in Caco-2 cells

**Figure 6:**
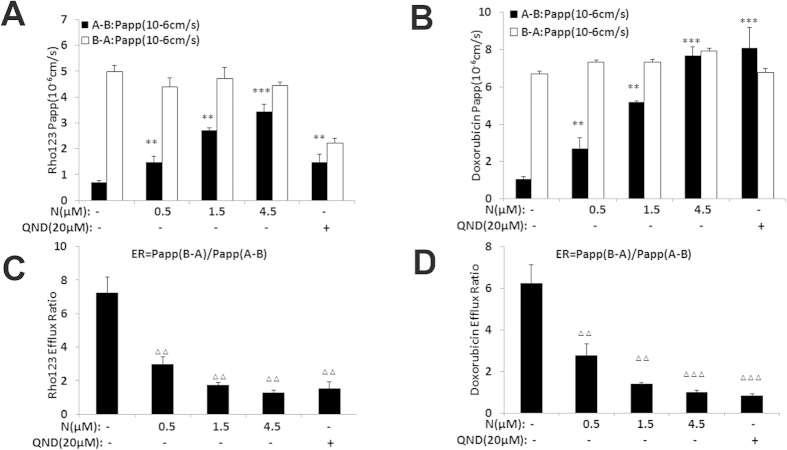
Nobiletin increased the adsorption and inhibit the efflux ratio of Rho123 and DOX in Caco-2 cells. Nobiletin increased the directional transport of Rho123 (5 μM) (**A**) and DOX (10 μM) (**B**) across Caco-2 cell monolayers. Nobiletin decreased the efflux ratio of Rho123 (5 μM) (**A**) and DOX (10 μM) in Caco-2 cell. □ AP → BL transport, ■ BL → AP transport. ΔΔ or **P < 0.01, ΔΔΔ or ***P < 0.001, significantly different from those obtained in the absence of nobiletin.

Human colorectal carcinoma Caco-2 cells are widely used as an *in vitro* model to predict human drug absorption and efflux activity of transporters^29^. To further confirm the effect of nobiletin on P-gp function, we determined the concentrations of the P-gp substrates Rho 123 and DOX in the presence or absence of nobiletin using the Caco-2 monolayer model. Two hours after administration, the values of Papp (A-B) of Rho 123 (Fig. 6A) and DOX (Fig. 6B) were increased dose-dependently in the presence of nobiletin. Moreover, the efflux ratio (the ratio between the Papp from the basolateral (BL) to the apical (AP) side and that from the AP to the BL side) was decreased in a nobiletin-concentration-dependent manner. As shown in Fig. 6C,D, significant decreases ( >  = 70%) in the efflux ratio of Rho 123 and DOX were observed in the presence of nobiletin. Intriguingly, the inhibitory effect of nobiletin at 4.5 μM was stronger than that of QND (20 μM) which was used as positive control.

These results were in agreed with the notion that nobiletin increased Rho 123, DOX, and Flutax-2 accumulations in the resistant ABCB1-overexpressing cells by inhibiting ABCB1 transporter.

### Nobiletin activates the ATPase activity of ABCB1

**Figure 7:**
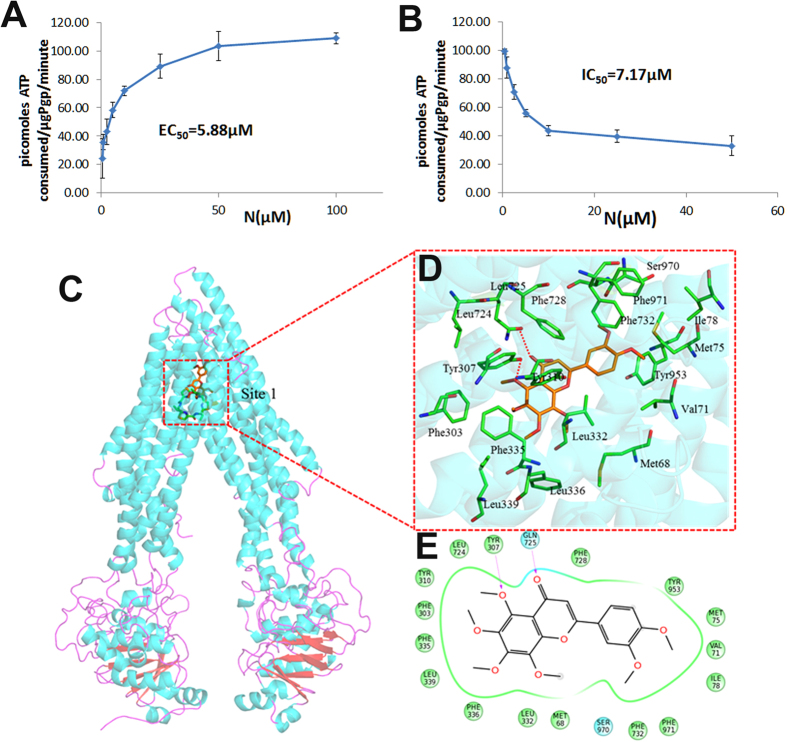
Mechanism studies for the inhibition effect of nobiletin on ABCB1 transporter. (**A**) EC_50_ measurements for stimulating P-gp ATPase activity by nobiletin; (**B**) IC_50_ measurements for inhibiting 200 μM verapamil-stimulated P-gp ATPase activity. Luminescence was read on a luminometer and data was analyzed as described in Material and Method. (**C**) Docking analysis of nobiletin with human ABCB1 homology model. The binding poses of QZ59-RRR (PDB: 4M2S) (green) and nobiletin (orange) are shown in site 1. (**D**) The interactions between nobiletin and the surrounding residues. The red dotted line represents hydrogen bond between atoms. (**E**) A two dimensional interaction sketch between nobiletin and its binding site residues of human ABCB1 is shown. Residues are shown as colored bubbles, cyan indicates polar and green indicates hydrophobic residues.

The efflux function of ABCB1 has a close relationship with ATP hydrolysis. Therefore, we measured ABCB1-mediated ATP hydrolysis with different concentrations of nobiletin. As shown in Fig. 7A, nobiletin stimulated the ATPase activity of ABCB1 in a dose-dependent manner, with EC_50_ of 5.88 μM and a maximal stimulation of 3-fold of the basal activity, suggesting that nobiletin affected the ATPase activity of ABCB1 and might interact at the drug-substrate-binding site as a substrate of ABCB1.

To characterize inhibition effect of nobiletin on ABCB1 ATPase activity, we also examined the effects of nobiletin on verapamil stimulated ABCB1 ATPase activity. Verapamil is sometimes referred as an ABCB1 inhibitor because, as a substrate for transport, it inhibits ABCB1 activity with other substrates by interfering with their transport in a competitive mode. Figure 7B showed the reduction of 200 μM verapamil-stimulated ATPase activity by nobiletin with an IC_50_ value of 7.17 μM, indicating nobiletin is an ABCB1 ATPase inhibitor.

### Nobiletin does not affect the expression of ABCB1

The reversal of ABCB1-mediated MDR can be achieved either by reducing ABCB1 expression or by inhibiting the function of ABCB1 transporter. Therefore, we investigated the effect of nobiletin on the expression of ABCB1 at both mRNA and protein level. At the selected concentrations used in the reversal assays, nobiletin did not significantly alter the mRNA expression of *MDR1* (Fig. 4A) or protein level of ABCB1 (Fig. 4B) in A2780/T cells. These findings revealed that the reversal effect of nobiletin was not due to the inhibition of ABCB1 expression. Therefore, the mechanism of sensitization of ABCB1-overexpressing MDR cells by nobiletin could be the inhibition of ABCB1 transporter function leading to an increase in intracellular accumulation of chemotherapeutic drugs.

### Molecular docking simulation of nobiletin within the drug binding cavity of ABCB1

To understand the binding mechanism of nobiletin to homology model of human ABCB1 at molecular level, we performed glide docking using ABCB1-QZ59-RRR (site-1), ABCB1-QZ59-SSS (site-2), ABCB1-verapamil (site-3), and a site common to above three sites (site-4) and an ATP binding site. According to the docking result, the pose of nobiletin was only accommodated to site 1 with Docking score (Kcal/mol) of −9.216 (Fig. 7C). There were no pose suitable for nobiletin to other three sites. Thus, site 1 was the only rational site for nobiletin. As shown in Fig. 7D, the binding site of nobiletin was partially superposed with the binding site of QZ59-RRR known as site 1.The ring-a of nobiletin substituted with four methoxyl groups was mainly engaged in hydrophobic contacts with Tyr307, Phe303, Tyr310, Phe335, Leu339, Leu336, Leu332. The methoxy of ring-a and the carbonyl of ring-b formed hydrogen bonds with Tyr 307 and Gln725 respectively, which also appeared in the binding of vardenafil and tadalafil. As for ring-c, the hydrophobic contacting with Phe732, Phe971, Ser970, Ile78, Met75, Tyr953, Val71 kept the conformation stable (Fig. 7E).

### Nobiletin-PTX combination inhibit the AKT/ERK and Nrf2 pathway

Moreover, nobiletin was reported to inhibit phosphorylation of AKT and phosphorylation of ERK2 in HGF-treated liver cancer HepG2 cells^30^. Considering the activation of PI3K/AKT and MAP kinase/ERK pathways in resistance MDR cancer cells^9^,^10^, hence, we examined the effect of nobiletin on the expression of total and phosphorylated AKT and ERK in A2780/T cells. After treatment with PTX and nobiletin for 48 h, there was significant inhibitory effect on phosphorylated AKT and ERK, but not on total AKT and ERK (Fig. 4B), indicating the inhibition of PI3K/AKT and MAP kinase/ERK pathways by the combination treatment. Moreover, there was a significant decrease in the phosphorylated AKT and ERK level after treatment with 50 μM of nobiletin (Fig. 4B) which was consistent with the published literature^30^,^31^. However nobiletin alone at reversal concentrations had no effect on the expression of total and phosphorylated AKT and ERK (supplementary Fig. S4). These results indicated that enhanced cytotoxic response by co-treatment with nobiletin and PTX in ABCB1 overexpression MDR cancer cells was associated with inhibition of PI3K/AKT and MAP kinase/ERK pathways.

Nuclear factor E2-related factor 2 (Nrf2) is a transcription factor that upregulates expression of a number of genes to combat oxidative and electrophilic stress. Recent studies reveal that activation of Nrf2 enhances chemo-resistance^8^, whereas blockade of Nrf2 sensitizes a variety of cancer cells to chemotherapeutic drugs^32^,^33^,^34^. In this study, we observed a remarkably higher level of Nrf2 in A2780/T cells as compared to A2780 cells (Fig. 4C). Nobiletin in combination with PTX reduced the protein level of Nrf2 in a dose-dependent manner, while nobiletin alone at reversal concentrations had no effect on the expression of Nrf2 (supplementary Fig. S4). These results clearly demonstrated that the reversal effect of nobiletin to PTX was related with the inhibition of Nrf2.

### Nobiletin-PTX combination increase the cellular accumulation of nobiletin

To clarify the strong synergistic cytotoxic effect of the nobiletin-PTX combination, we also evaluated the effect of co-treatment of nobiletin-PTX on the intracellular level of nobiletin by LC-MS/MS method. Both data acquisition parameters for MRM mode (multiple-reaction monitoring) of nobiletin and naringenin (internal standard) were optimized with MassHunter Optimizer software on triple quad mass spectrometer. Using this developed method, the intracellular concentrations of nobiletin in A2780/T cells after 48 h treatment were evaluated and shown in Supplementary Table 3. We noticed that the intracellular concentration of nobiletin in A2780/T cells was increased from 82.28 ± 2.36 ng/mg protein in single treatment of 9 μM nobiletin to 118.41 ± 4.06 ng/mg protein (increased about 40%) in combination with 0.94 μM PTX. Thus, the observed strong synergistic cytotoxic effect of the nobiletin-PTX combination maybe related with the intracellular accumulation of nobiletin. Furthermore, the dose dependent inhibition of activated AKT and ERK with combination of serial concentrations of nobiletin with PTX (0.94 μM) in Fig. 4B maybe also resulted from the increased intracellular concentration of nobiletin.

## Discussion

Traditional chemotherapy drugs such as PTX remain the cornerstone of tumor therapy, but the occurrence of drug resistance has been a major obstacle leading to the failure of treatment. However, up to now, none of the compounds from the three generation of MDR modulators have been approved for clinical use^13^,^35^,^36^,^37^. Currently, discovering more efficacious, non-toxic and less expensive compounds from natural products to reverse MDR is gaining increasing interest.

Nobiletin was reported to inhibit the growth of several prostate cancer cell lines with IC_50_ values around 100 μM^20^,^21^,^22^ and potential P-gp inhibition effect at 20 μM or 50 μM^23^,^24^,^25^. In this study, nobiletin at non-cytotoxic concentrations (0.5-9 μM) significantly increased the sensitivity of ABCB1 overexpressing A2780/T, and A549/T cell lines to chemotherapeutic agents such as DOX, PTX, docetaxel and dounorubicin, whereas it could not potentiate the effect of these substrate drugs on parental cells. A significant decrease in the IC_50_ value of PTX (with reversal fold of 432) was observed for the first time after co-treatment with PTX and nobiletin together. In addition, combination studies indicated that nobiletin was a very strong synergist for enhancing the anti-tumor effect of PTX in MDR cancer cell lines, and predicted that 0.286 μM of nobiletin could bring a 200-fold decrease on the ED_50_ of PTX. Importantly, the concentrations of nobiletin used in this study were lower than the maximal plasma concentration (22.5 μM) obtained in *in vivo* pharmacokinetic study of nobiletin^26^. Therefore, nobiletin might potentially be a MDR modulator.

To investigate the mechanism of the powerful reversing effect of nobiletin to PTX in ABCB1 overexpression cancer cells, we examined the effects of nobiletin on the drug transport activity of ABCB1 and the expression of P-gp protein. In accordance with the cytotoxicity assay, nobiletin remarkably enhanced the intracellular accumulation of DOX and flutax-2 in drug resistant cells but not the parental sensitive cells, indicating nobiletin might affect the ABCB1 function. Furthermore, we demonstrated that nobiletin could inhibit the efflux activity of ABCB1 transporter in Caco-2 monolayer cell model. However, nobiletin did not affect the ABCB1 expression at both mRNA and protein levels at the reversal concentrations. These results suggested that nobiletin modulated the transporter activity of ABCB1 thus enhanced the intracellular drug concentration resulting in the appearing cytotoxicity of chemotherapeutic agents.

To further investigate the interaction between nobiletin and P-glycoprotein (ABCB1) transporter, the effect of nobiletin on the ATPase activity was examined. As energy used by ABCB1 transporter comes from ATP hydrolysis, several studies have demonstrated that P-gp inhibitors could stimulated the ATPase activities either in a dose-dependent manner or in a bidirectional way^38^. We found that the activity of ATPase was stimulated by nobiletin in a concentration dependent manner indicating that nobiletin might potentially be a substrate of ABCB1. Moreover, verapamil-stimulated ATPase activity was reduced by nobiletin. Therefore, it may competitively bound to the substrate-binding site of ABCB1, leaving little place for other agents to bind to the transporter, which resulted in decreased activity of ABCB1 transporter. Further docking analysis was carried out with human ABCB1 homology model to clearly illustrate the molecular interaction between human ABCB1 and nobiletin. Our results reveal the predicted binding conformation of nobiletin within the large hydrophobic drug binding cavity (site-1) of human ABCB1 with the major contributions of hydrophobic interactions. In addition, we also noticed that the combination of nobiletin-PTX could increase the intracellular accumulation of both nobiletin and PTX. Taken together, we concluded that nobiletin may inhibit the activity of ABCB1 transporter by competitively binding to the substrate-binding site (site-1) of ABCB1 transporter leading to the increased intracellular concentration of both nobiletin and ABCB1 substrate.

Besides the acquired resistance of cancer cells to various chemotherapeutic agents via accelerated efflux of anticancer agents, there are other mechanisms by which these cells become resistant to these drugs. Pervious preclinical and clinical evidences suggested that the PI3K/AKT, MAPk/ERK and Nrf2 signaling pathways were associated with resistance to multiple chemotherapeutic drugs^9^,^39^,^40^. Inactivating the AKT/ERK and Nrf2 signaling pathways renders MDR cancer cells more sensitive to drugs such as paclitaxel, doxorubicin, 5-fluorouracil, *etc.*^8^,^10^. Therefore, we subsequently studied the relationship between the action of nobiletin on the activated AKT/ERK and Nrf2 pathways and reversal of MDR resistance. Here, we observed constitutive activation of the AKT/ERK and Nrf2 pathways in A2780/T cells, which was not observed in A2780 cells. Moreover, the nobiletin-PTX combination reduced phosphorylated AKT/ERK level and Nrf2 expression, which indicated that the inhibition of AKT/ERK and Nrf2 also account for the sensitizing effect of nobiletin in MDR-cancer cells. These may be not only helpful for illustrating the multiple mechanisms behind the reversal effect of nobiletin, but also helpful for explaining the reversal effect of nobiletin to 5-fluorouracil which is not a P-gp substrate. Literatures have demonstrated that the inhibition of Nrf2 expression could be through PI3K/AKT and ERK signaling pathway^33^. Thus, mechanistically, nobiletin sensitizes the MDR cancer cells to chemotherapeutic agents could be through significantly reducing Nrf2 expression by down-regulating the PI3K-Akt and ERK pathways. Moreover, all these results support for our hypothesis that the powerful MDR-reversal properties of nobiletin to PTX and other chemotherapeutical agents observed in this study would be the results of modulation of multiple proteins and signaling pathways related to the MDR. Research in other pathways related to significant reversal effect of nobiletin should be actively pursued to explain the complex mechanisms of cancer multidrug resistance.

In conclusion, this study provided the first evidence that nobiletin significantly reversed ABCB1 mediated MDR by inhibiting the efflux function of ABCB1 transporter and suppressing the chemoresistance related AKT/ERK/Nrf2 pathways. As a very strong synergist, nobiletin promoted cell apoptosis as well as G2/M cell cycle arrest induced by PTX and reduced the EC_50_ value of PTX. However, the reversal effect of nobiletin was independent of ABCB1 expression. Given the broad-spectrum organ safety of nobiletin demonstrated in animal models *in vivo*^41^,^42^,^43^, our present work suggests that nobiletin, as combination therapy, may be a good candidate for studies *in vivo* to reverse ABCB1-medicated drug resistance in cancer therapy.

## Methods

### Reagents

Nobiletin was purchased from Dalian Meilun Biology Technology Co., Ltd, and the structure and purity was confirmed by LC-MS in our lab. Flutax-2 was purchased from Life Technologies. Paclitaxel (PTX) and doxorubicin (DOX), verapamil (Vrp), quinidine (QND), 5-fluorouracil, docetaxel, daunorubicin, dimethyl sulfoxide (DMSO), RNase A, leupeptin, aprotinin, phenyl methyl sulfonyl fluoride, Triton X-100, propidium iodide (PI) and other chemicals were purchased from Sigma-Aldrich (St. Louis, MO). Stock solutions of nobiletin (40 mM), DOX (40 mM) and PTX (80 mM) were prepared DMSO and appropriate working concentrations were prepared in cell culture medium immediately before use. The RPMI 1640 medium, fetal bovine serum, penicillin and streptomycin were obtained from Life Technologies Inc. (Grand Island, NY). ERK 1/2 and actin antibodies were purchased from Santa Cruz Biotechnology, USA; P-gp and P53 antibodies were purchased from Calbiochem and Abcam; other antibodies such as AKT, P-AKT, and P-ERK1/2 were purchased from Cell Signaling Technology, Inc.

### Cell culture

Human ovarian cancer cells A2780 and its PTX-resistant cell line A2780/T, human non-small cell lung cancer (NSCLC) A549 and its PTX-resistant cell line A549/T were generously provided by Professor Zhi-Hong Jiang (Macau University of science and technology, Macau). Cells were grown as monolayers in RPMI-1640 medium supplemented with 10% fetal bovine serum (GIBCO, Paisley, Scotland) at 37 °C in a humidified 5% CO_2_ atmosphere. The indicated concentration of paclitaxel (0.94 μM) was added to the culture medium to maintain drug resistance for A2780/T and A549/T. The mRNA level of P-gp didn’t changed significantly after grown in drug- free culture medium for 10 days for both resistant cell lines. The human colon carcinoma cell line Caco-2 was purchased from the ATCC, and cells at passage numbers 25–35 were used for the assays.

### Cell cytotoxicity assay

Sulphorhodamine B (SRB) assays were used for cell density determination, based on sensitive measure of total cellular protein, which perform similarly compared with other proliferation assays such as MTT assay^44^. Briefly, cells were seeded into flat bottomed 96-well plates at an initial density of 7.5 × 10^3^ per well before treatment. Cells were exposed to varying concentrations of nobiletin (9, 4.5, 1.5 and 0.5 μM) and combined them with varying concentrations of PTX (1 μM to 0.03 nM with 3.16 fold diluted, 10 μM to 0.3 nM with 3.16 fold diluted, 100 μM to 3 nM with 3.16 fold diluted respectively) to test whether this combination can enhance the growth inhibition of MDR cancer cells. After removing the medium, cells were fixed in 10% trichloroacetic acid for 1 h at 4 °C and then washed with water five times. 0.4% SRB dissolved in 1% v/v acetic acid was added and incubated 30 min for staining. The cells were quickly washed with 1% acetic acid and left to dry overnight. The protein bound SRB was solubilized by adding 200 μl 10 mM Tris buffer per well and was measured at wavelengths 490 nm using a plate reader (Spectra MAX 250; Molecular Devices, Sunnyvale, CA). The degree of resistance was estimated by comparing the IC_50_ (concentration of 50% inhibition) for the MDR cells to that of parent sensitive cells, while, the degree of reversal of MDR was calculated by dividing the IC_50_ for cells with the chemotherapeutic drugs in the absence of nobiletin by that obtained in the presence of nobiletin.

### Colony formation assay

For the colony formation assays, A2780/T cells (1200 cells/well) in 6-well plates were treated with culture medium (containing 0.94 μM PTX) or with nobiletin in different concentration (containing 0.94 μM PTX) for 8 days. Subsequently, the cells were fixed with 70% ethanol and stained with crystal violet (0.5% in ethanol). The plates were rinsed with phosphate buffered saline (PBS), and the colony numbers were counted using the software of Quantity one-Colony counting.

### Cell cycle analysis

A2780/T cells were harvested after 24 hours, 48 hours, or 72 hours treatment and washed twice with ice-cold PBS. The cells were fixed and permeabilized with 70% ice-cold ethanol overnight at 4 °C or 2 h at −20 °C. After one additional wash in PBS, cells were stained with a staining solution containing PI (50 μg/ml) and RNase A (200 μg/ml) for 30 min at room temperature. They were then pelleted, washed and suspended in PBS to a final concentration of 1 × 10^6^/ml and analyzed by flow cytometry BD FACS Aria (BD Biosciences, San Jose, CA).

### Apoptosis analysis by flow cytometry

After treatment, 1 × 10^6^ cells were collected, washed and suspended in 100 μl of binding buffer (10 mM N-2-hydroxyethylpiperazine-N,-2-ethanesulfonic acid/NaOH, 140 mM NaCl, 2.5 mM CaCl2, pH 7.4). Apoptotic cells were identified by double supravital staining with 5 μl recombinant FITC (fluorescein isothiocyanate) -conjugated Annexin-V and 5 μl PI (50 μg/ml). The cells were stained for 15 min at room temperature in the dark, and analyzed by fluorescence-activated cell sorting cater-plus flow cytometry. Data acquisition and analysis were performed in BD FACS Aria with FlowJo software.

### Drug combination assay

The synergistic therapeutic effect for the combination of nobiletin and PTX was evaluated using the Chou-Talalay Method^28^. “Combination index” (CI) was calculated by this method to quantitatively depict synergism (CI < 1), additive (CI = 1), or antagonism (CI > 1) effect. Briefly, drug resistant A2780/T cells were exposed to a serially diluted mixture of nobiletin (IC_50_ = 31.62 μM) and PTX (IC_50_ = 2.51 μM) for 48 hours. The 2-fold serial dilution with several concentration points above and below its IC_50_ value was used for evaluating cytotoxicity of combination by SRB method as above description. With the use of CalcuSyn software v. 2.1 (Bio-soft), synergy is further refined as synergism (combination index = 0.3–0.7), strong synergism (combination index = 0.1–0.3), and very strong synergism (combination index < 0.1)^45^.

### Intracellular accumulation of doxorubicin and flutax-2

For fluorescence microscopy observation, A2780 or A2780/T cells (5 × 10^6^) were cultured on the cover glass (ISO LAB 20 × 20 mm). DOX (5 μM), or flutax-2(1 μM) alone or in combination with nobiletin (4.5 μM) was added and incubated for 8 h. After treatment, cells were fixed in 4 wt% formaldehyde (Sigma-Aldrich). Nuclear DNA was stained with 1 μg/mL blue-fluorescent DAPI (1 mg/mL in H_2_O stock solution; Invitrogen D1306). One drop of fluorescent preservation solution (fluorsave reagent, CALBIOCHEM) was added before observation. Imaging was carried out for comparing the intracellular accumulation of DOX and flutax-2 with a Fluorescence Microscopy (Leica DM2500, Leica, Geman).

For flow cytometry analysis, flutax-2(1 μM) and DOX (5 μM) was added to A2780 or A2780/T cells and incubated with or without nobiletin (4.5 μM) for 8 h. Cells were detached, re-suspended in 500 μl of PBS after washed twice with cold PBS, and analyzed by flow cytometry (BD FACS Aria, BD Biosciences, San Jose, CA). Excitation and emission wavelengths (nm) used for DOX and flutax-2 were as follows: 480 to 585; and 496 to 524. Quinidine (QND, 20 μM), a known ABCB1 inhibitor, was used as a positive control.

### Transport assay in Caco-2 monolayer model

The Caco-2 cell line was seeded on Millipore Millicell plates and formed a confluent monolayer over 21 days prior to the experiment. The integrity of the cell monolayers was checked by measuring the transepithelial electrical resistance (TEER) before and after the transport experiments using a WPI EVOM2 Epithelial voltohmmeter fitted with STX2 chopstick electrodes (World Precision Instruments, Sarasota, FL, USA). On day 21, the transport assay included apical-to-basolateral (A → B) and basolateral-to-apical (B → A) transport rate determinations for rhodamin123 (5 μM) and DOX (10 μM) in Caco-2 cell line was carried out over a 2 hour time period^29^. Briefly, samples (100 μL) were collected from apical/basolateral side of Caco-2 cell monolayer at predetermined times of 30, 60, 90, and 120 min, and immediately detected for the fluorescence intensity in 96 well black plate (Corning; Cat. 3603) using a microplate reader (infinite M200 PRO, TECAN, Switzerland). For inhibition studies, bidirectional transport of target compound was conducted in Caco2 cell monolayer with nobiletin added in both apical and basolateral chambers. Quinidine (QND) was used as potent control inhibitors of P-gp.

The apparent permeability coefficients (Papp) were calculated as


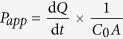


where dQ/dt (mM/sec) is the rate of permeation of compound across the cells, C_0_ (mM) is the donor compartment concentration at time zero and A (cm^2^) is the area of the cell monolayer. The decrease in Efflux Ratio (ER = Papp (B to A)/Papp (A to B)) in the presence of nobiletin and putative inhibitor QND was determined to assess their relative inhibitory potency to transporter P-gp.

### ABCB1 ATPase activity assay

The impact of nobiletin on P-gp ATPase activity was estimated by Pgp-Glo™ assay systems (Promega, USA). The inhibitory effects of nobiletin were examined against a verapamil-stimulated ABCB1 ATPase activity. Sodium orthovanadate (Na_3_VO_4_) was used as an ABCB1 ATPase inhibitor. Following manufacture’s instruction, 0.25 mM Na_3_VO_4_, 0.5 mM verapamil, or nobiletin in various concentrations were incubated with assay buffer, 25 μg recombinant human ABCB1 membranes and 5 mM MgATP at 37 °C for 40 min. For examination the inhibitory effects of nobiletin against verapamil-stimulated P-gp ATPase activity, then 200 μM verapamil was added with nobiletin together. Luminescence was initiated by ATP detection buffer. The plate (white opaque 96-well, corning, USA) was further incubated at room temperature for 20 min to develop luminescent signal, and was read with luminometer (infinite M200 PRO, TECAN, Switzerland). The changes of relative light units (ΔRLU) were determined by comparing Na_3_VO_4_-treated samples with nobiletin only or nobiletin and verapamil combination-treated samples, and hence, the ATP consumed was calculated by comparing to a standard curve.

### RT-PCR analysis

RT-PCR was performed to evaluate MDR1 mRNA expression. mRNA from cell lysates were purified by binding to poly(dT) magnetic beads (Life technologies) and reverse transcribed by using SuperScript II (Life technologies). Standard quantitative RT-PCR was performed in duplicates at least two to three times by using SYBR Green (Molecular Probes) protocols on the ViiA™ 7 Real-Time PCR System (Life technologies). The primer sequences: 5′-GAGAGATCCTCACCAAGCGG-3′ and 3′-CGAGCCTGGTAGTCAATGCT-5′ for MDR1, and 5′-AGAAGGCTGGGGCTCATTTG-3′ and 3′-AGGGGCCATC-CACAGTCTTC-5′ for control gene eukaryotic translation initiation factor (TIF)^44^. RT-PCR data were normalized by measuring average cycle threshold (Ct) ratios between candidate genes and control gene TIF.

### Western blot analysis

The total cellular samples were harvested and rinsed twice with ice-cold PBS buffer. Cell extracts were lysed in RIPA buffer (50 mM Tris (pH 7.4), 150 mM NaCl, 1% Triton X-100, 1% sodium deoxycholate, 0.1% SDS, sodium orthovanadate, sodium fluoride and EDTA) containing protease inhibitor cocktails (Roche Life Science, USA). Protein concentration was determined using the BCA protein assay kit. Equal amounts of cell lysates were resolved by SDS-PAGE and subsequently electrophoretically transferred onto PVDF membranes (Millipore, Darmstadt, Germany). After blocking in tris-buffered saline containing 0.1%of Tween20 (TBST) with 5% (w/v) skim milk (Nestle Carnation, New Zealand) for 2 h at room temperature, the membranes were incubated with primary and secondary antibodies and subsequently visualized with an enhanced chemiluminescence detection kit (Thermo Scientific™ SuperSignal™ West Pico Chemiluminescent Substrate, USA). β-Actin was used as the loading control for the experimental data analysis.

### LC-MS/MS conditions

Analysis of cellular fractions were performed on an AB Sciex 4000 QTRAP® Mass Spectrometer(SCIEX, USA) connected with an Waters ACQUITY UHPLC system (Waters Co., USA), using a X Bridge^TM^ BEH C18 analytical column (1.7 μm 2.1 × 50 mm; Waters, Torrance, CA). A linear gradient mobile phase composed of 0.1% formic acid water (solvent A) and acetonitrile (solvent B) was mixed according to the following gradient program: 0–5 min (20–55% B); 5–12 min (55–65% B); 12–13 min (65–20% B). The injection volume was 10 μl and flow rate was 0.4 ml/min.

For our final method, MS parameters were as follows: Ionspray voltage, 4000 V; source temperature, 400 °C; Gas 1/2, 30 psi; curtain gas, 30 psi; collision energy, 30 V; dwell time, 100 ms. Precursor-to-product ion transition of *m/z* 403.3 → 373.2 for nobiletin and *m/z* 271.0 → 150.8 for naringenin (internal standard) were used for multiple reaction monitoring (MRM).

### Preparation samples for LC-MS/MS analysis

Briefly, a 200 μl aliquot of the sample was mixed with 800 μl acetonitrile solution containing 25 ng/ml naringenin as IS by vortexing for 1 min. The mixture was centrifuged at 13,000 rpm for 15 min at 4 °C. A 800 μl volume of the supernatant was evaporated to dry using Organomation’s MICROVAP Microplate Nitrogen Evaporators (Berlin, MA USA). Then the residue was dissolved with 100 μl 20% acetonitrile solution and vortexed for 1 min before centrifugation at 13,000 rpm for 15 min at 4 °C. The supernatant (10 μl) was injected into the LC-MS/MS system for analysis.

The protein content of each fraction was determined using BCA protein assay kit (Bio-Rad, Philadephia, USA). The concentration of nobiletin in each subcellular fraction was determined by LC-MS/MS, and then normalized with protein amount.

### Molecular modeling – ABCB1

In order to figure out the exact binding site for nobiletin, we used homology modeling and molecular docking to study the interaction between human P-Glycoprotein and nobiletin.

Human P-glycoprotein (ABCB1) was thought to have four sites interacting with the inhibitors^46^,^47^, so we rebuilt the four sites using Prime v2.1 in Maestro 9.0 (Schrodinger, Inc., New York, NY, 2009). The 3D structures of ABCB1 from the mouse was selected as the templates: The complex structure cocrystallized with QZ59-RRR (PDB: 4M2S) for site 1, the complex structure cocrystallized with QZ59-SSS (PDB: 4M2T) for site 2, the apo structure (PDB: 3G5U) for site3 and site 4. The ligands from the complex templates were retained and used to define the site 1 and site 2 in the homology structures. The site 3 was defined by residues contributing to verapamil binding and the site 4 was defined by two residues which were common to the other three sites^48^.

All the docking calculations for four sites were performed in the Induced Fit Docking module (Schrodinger, Inc., New York, NY, 2009) and the pose was ranked by the XP mode of Glide program v5.5 (Schrodinger, Inc., New York, NY, 2009). Then we selected the pose with the highest docking for further conformational analysis.

### Statistical analysis

All experiments were repeated at least three times and the data were presented as the mean ± SD unless noted otherwise. Statistical analysis was carried out using Student’s t-test or one-way analysis of variance with Microsoft Excel 2010, and the level of significance was set at a P value of < 0.05(*), <0.01 (**) or <0.001(***).

## Additional Information

**How to cite this article**: Ma, W. *et al.* Nobiletin enhances the efficacy of chemotherapeutic agents in ABCB1 overexpression cancer cells. *Sci. Rep.* **5**, 18789; doi: 10.1038/srep18789 (2015).

## Supplementary Material

Supplementary Information

Supplementary Information

## Figures and Tables

**Table 1:** Nobiletin reverses the ABCB1-mediated drug resistance to 5-fluorouracil, docetaxel and doxorubicin in A2780/T cells.

A2780/T		
Drug	IC_50_ ± SD (μM)	fold reversal
Paclitaxel	2.67 ± 0.22	1.00
+0.5 μM N	0.90 ± 0.15***	3.00
+1.5 μM N	0.15 ± 0.02***	18.47
+4.5 μM N	0.02 ± 0.004***	163.46
+9 μM N	0.006 ± 0.0002***	432.98
Docetaxel	17.9 ± 2.89	1.00
+0.5 μM N	8.97 ± 1.46*	2.05
+1.5 μM N	3.57 ± 0.58**	5.01
+4.5 μM N	1.13 ± 0.18***	15.84
Doxorubicin	5.97 ± 0.49	1.00
+0.5 μM N	3.77 ± 0.30**	1.58
+1.5 μM N	1.89 ± 0.16***	3.16
+4.5 μM N	1.13 ± 0.18***	5.39
+9 μM N	0.38 ± 0.04***	15.92
Daunorubicin	11.91 ± 0.97	1.00
+0.5 μM N	9.46 ± 0.77	1.26
+1.5 μM N	4.49 ± 0.73*	2.70
+4.5 μM N	0.89 ± 0.15**	13.58
5-Fluorouracil	159.19 ± 18.30	1.00
+0.5 μM N	132.2 ± 23.78	1.21
+1.5 μM N	79.78 ± 9.18*	1.99
+4.5 μM N	26.14 ± 1.77*	6.08

Cell growth was determined using the SRB assay. The data are representative of three different experiments and are shown as mean ± SD (n = 3). *P < 0.05, **P < 0.01, ***P < 0.001, means significantly different from the control group in the absence of nobiletin.
