# Supplementary material for: Nobiletin enhances the efficacy of chemotherapeutic agents in ABCB1 overexpression cancer cells
Source: Sci Rep. 2015 Dec 22;5:18789. doi: 10.1038/srep18789 (PMC4686932; doi:10.1038/srep18789)
Supplement: Supplementary Information [file srep18789-s1.pdf]

Nobiletin enhances the efficacy of chemotherapeutic agents in ABCB1 overexpression cancer cells

Wenzhe Ma, Senling Feng, Xiaojun Yao, Zhongwen Yuan, Liang Liu, Ying Xie*

Supplementary Information

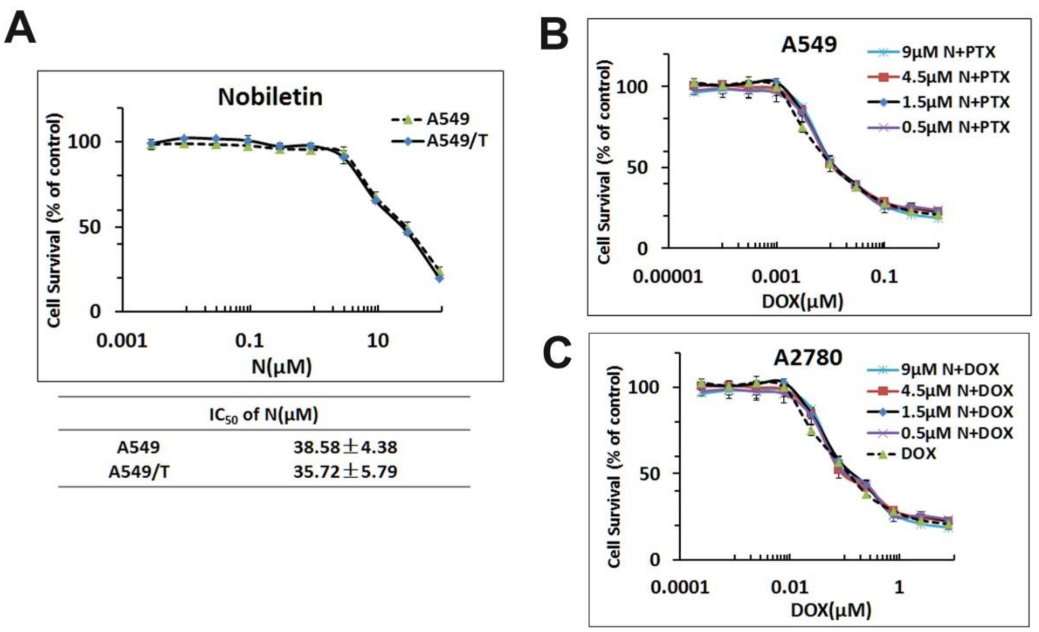

Supplementary Figure S1. Nobiletin have no reversal effect on the paclitaxel sensitivity cells. **(A)** Cytotoxicity of nobiletin alone in pairs of A2549/T or A549 cells. Nobiletin did not affect the IC_{50} of paclitaxel in sensitive cancer cells A2780 **(B)** and A549 **(C)**. Cells were treated with the indicated drugs for 48 hours and subjected to SRB assay. **, $P<0.01$., ***, $P<0.001$., Student's t-test (n = 3) or one-way ANOVA (n = 3).

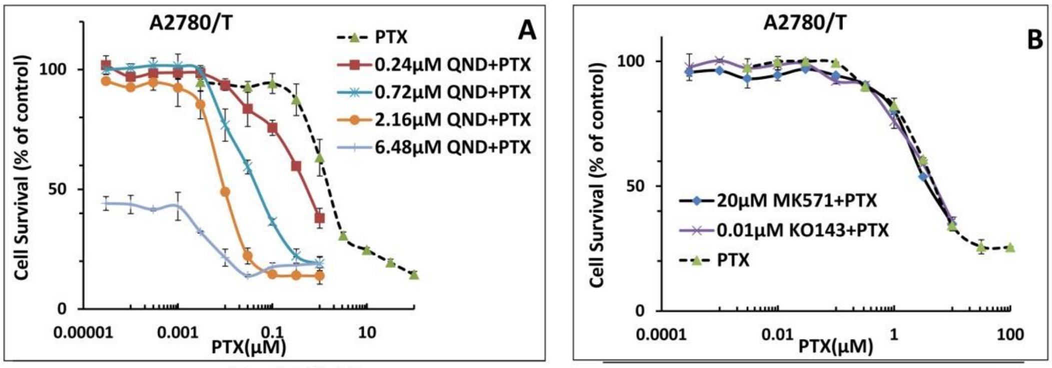

Drug	IC$_{50}$ of PTX(μM) A2780/T	Fold reversal
PTX	1.94±0.22	1.00
0.24μM QND+PTX	0.60±0.05**	3.25±0.1
0.72μM QND+PTX	0.05±0.01***	43.15±1.91
2.16μM QND+PTX	0.01±0.003***	252.83±61.99
6.48μM QND+PTX	/	/

Drug	IC$_{50}$ of PTX(μM) A2780/T
PTX	4.69±0.45
0.01μM KO143+PTX	4.9±0.16
20μM MK571+PTX	3.89±0.13

Supplementary Figure S2. The effect of transporter inhibitor on the paclitaxel sensitivity of resistant cells. **(A)** Quinidine (MDR1 inhibitor) but not **(B)** MK571(MRPs inhibitor) or KO143 (BCRP inhibitor) reduces the IC$_{50}$ of paclitaxel in resistant cancer cells A2780/T. Cells were treated with the indicated drugs for 48 hours and subjected to SRB assay. **, P<0.01., ***, P<0.001., Student's t-test (n = 3) or one-way ANOVA (n = 3).

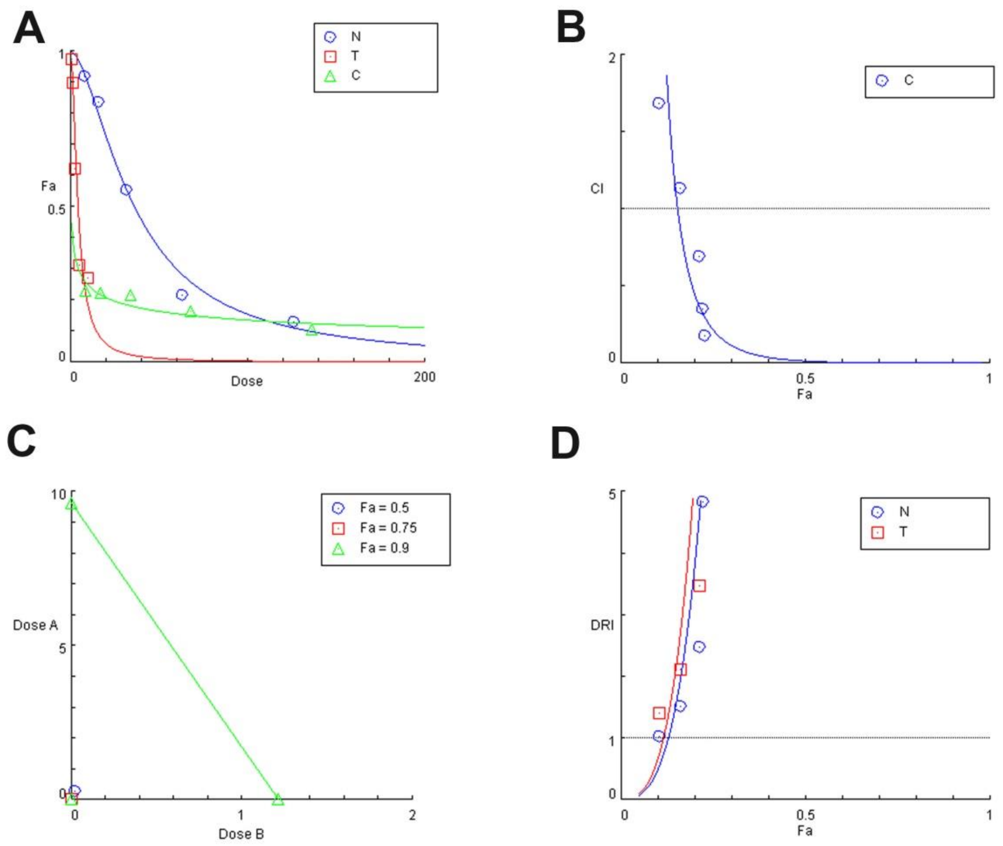

Supplementary Figure S3. The quantitative diagnostic graphics for the synergistic effect between nobiletin (N) and paclitaxel (T) generated by the computer simulation. **(A)**, the fraction affected (Fa)-Dose plot for nobiletin (N), paclitaxel (T) and combination (C). **(B)**, the Fa-CI plot (Chou-Talalay plot). **(C)**, the classic isobologram. **(D)**, the Fa-DRI plot (Chou-Martin plot) for the constant ratio combination design. CI < 1, Synergism; CI = 1, Additive; CI > 1, Antagonism. DRI > 1, Reduced dose and reduce Toxicity.

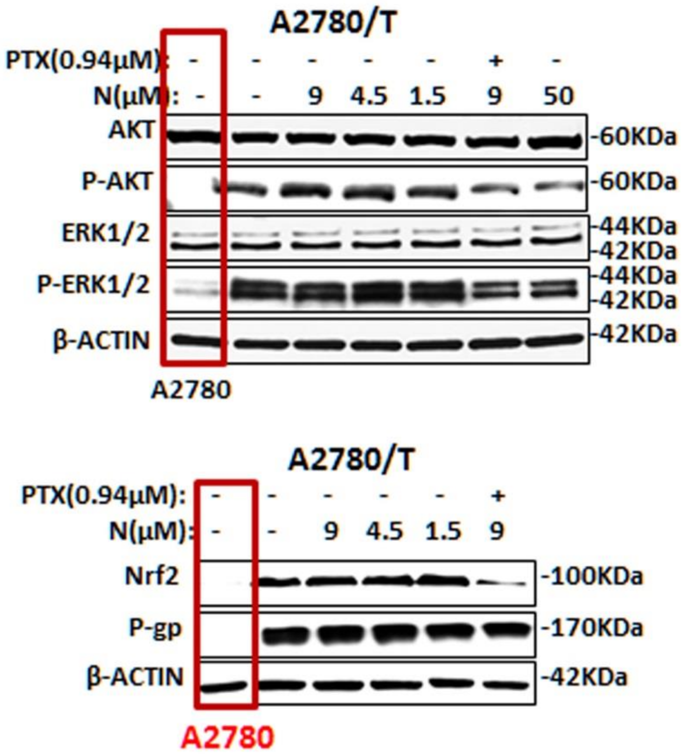

Supplementary Figure S4. Effects of nobiletin alone on ABCB1 expression and AKT/ERK/Nrf2 pathway. Equal amounts of total lysate were loaded and detected by Western blot. Nobiletin alone did not influence ABCB1, Nrf2, total and phosphorylation of AKT/ERK expression levels.

Supplementary Table S1 Nobiletin reverses the ABCB1-mediated drug resistance to Paclitaxel, 5-fluorouracil, docetaxel and doxorubicin in A549/T cells.

	A549/T	
Drug	IC50(μM)	Fold reversal
Paclitaxel	1.89±0.15	1.00
+0.5μM N	0.75±0.06*	2.51±0.01
+1.5μM N	0.34±0.02**	5.65±0.8
+4.5μM N	0.09±0.01***	22.33±3.6
+9μM N	0.03±0.004***	62.88±0.98
Docetaxel	13.36±1.09	1.00
+0.5μM N	8.43±0.69*	1.59±0.01
+1.5μM N	2.67±0.22**	5.05±0.82
+4.5μM N	0.71±0.11**	19.18±4.59
Doxorubicin	5.97±0.49	1.00
+0.5μM N	4.23±0.35	1.42±0.23
+1.5μM N	2.67±0.22*	2.25±0.37
+4.5μM N	1.19±0.10**	5.01±0.01
Daunorubicin	10.07±1.63	1.00
+0.5μM N	5.97±0.49*	1.68±0.14
+1.5μM N	2.84±0.46*	3.55±0.01
+4.5μM N	0.71±0.13**	14.17±0.04
5-Fluorouracil	133.57±10.86	1.00
+0.5μM N	106.10±8.63	1.26±0.01
+1.5μM N	66.95±5.44*	1.99±0.01
+4.5μM N	16.82±1.36**	7.94±0.02

Cell growth was determined using the SRB assay. The data are representative of three different experiments and are shown as mean ± SD (n = 3). *, P<0.05,**, P<0.01, ***, P<0.001, means significantly different from the control group in the absence of nobiletin.

Supplementary Table S2 The calculated CI for combination of nobiletin and paclitaxel as well as the simulated synergism dose at Fa 0.5 (ED_{50})

Data for Fa = 0.5	CI value	Dose N(μM)	Dose PTX(μM)
N		35.9574	
PTX			4.20446
N+PTX	0.01334	0.28572	0.02268

CI analyses of the effects of nobiletin in combination with paclitaxel are shown. The CI values were plotted as a function of the particular inhibitory effect. CI values <1 represent a synergistic combination, CI values equal to 1 are additive and CI values >1 represent antagonistic combinations. We conclude from the table that PTX was significantly reduced in nobiletin treated A2780/T cells.

Supplementary Table S3 The cellular concentration of nobiletin in A2780/T cells after treatment with single nobiletin or in combination of PTX 48hours (n=3).

Group	N Conc. ($\overline{X} \pm S.D.$ ng/mg protein) *
4.5μM N	44.87±2.57
4.5μM N+ Paclitaxel	92.86±7.96
9μM N	82.28±2.36
9μM N+ Paclitaxel	118.41±4.06
25μM N	138.01±5.73
50μM N	315.69±3.12

*Final intracellular concentration of nobiletin was expressed as $\overline{X} \pm S.D.$ ng/mg protein (n=3).
