# Supplementary material for: Nobiletin enhances the efficacy of chemotherapeutic agents in ABCB1 overexpression cancer cells
Source: Sci Rep. 2015 Dec 22;5:18789. doi: 10.1038/srep18789 (PMC4686932; doi:10.1038/srep18789)

Nobiletin enhances the efficacy of chemotherapeutic agents in ABCB1 overexpression cancer cells

Wenzhe Ma, Senling Feng, Xiaojun Yao, Zhongwen Yuan, Liang Liu, Ying Xie*

Supplementary Figure Legends Full-length blots for Figures 4B, 4C, and supplementary figure S4.

Relative to Figure 4B

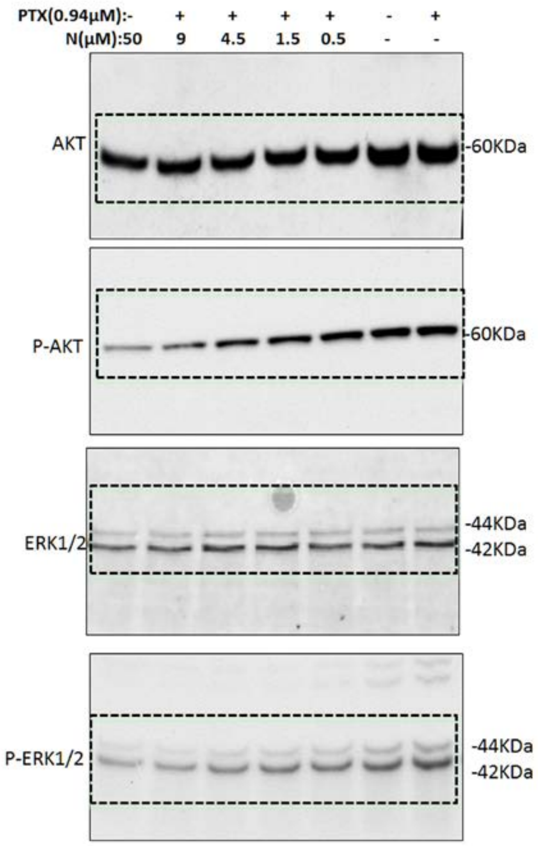

Relative to Figure 4B

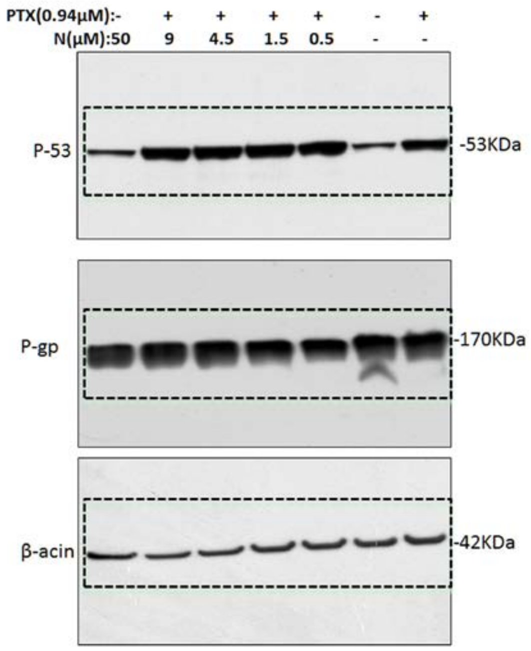

Supplementary Figure 4C

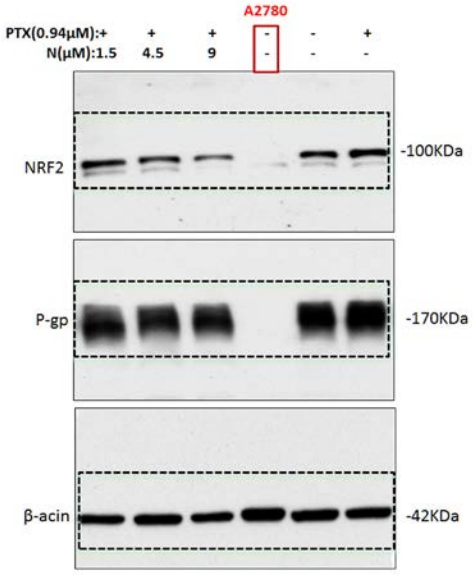

Relative to Supplementary Figure S4

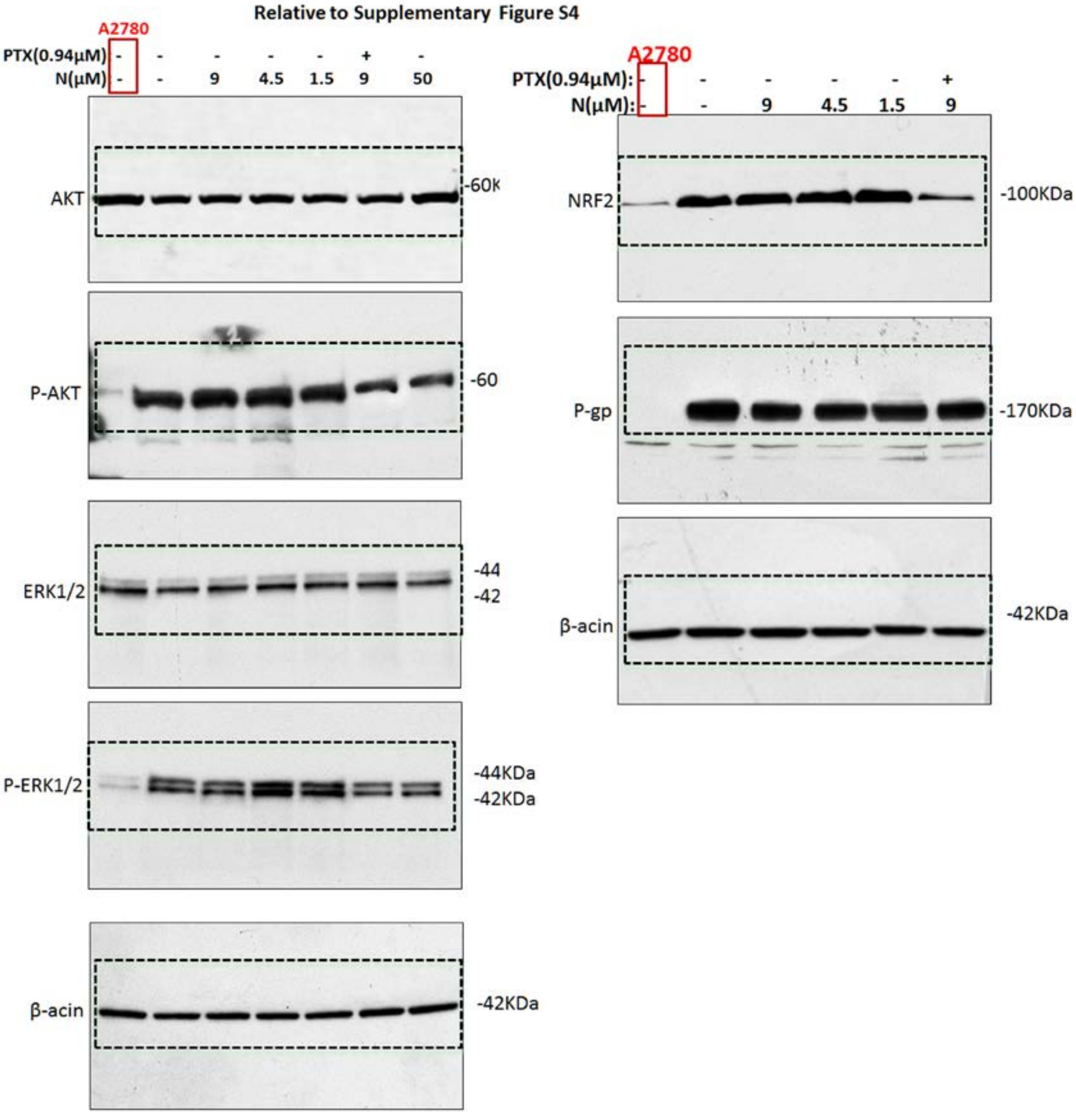

Supplement: Supplementary Information [file srep18789-s2.pdf]
